# Admission Inflammatory Blood Cell Ratios as Prognostic Markers of Functional Outcome After Aneurysmal Subarachnoid Hemorrhage: A Single-Center Retrospective Cohort Study

**DOI:** 10.3390/biomedicines14061186

**Published:** 2026-05-24

**Authors:** Renata Jabłońska, Robert Ślusarz, Agnieszka Królikowska, Karolina Filipska-Blejder, Magdalena Zając, Paweł Sokal

**Affiliations:** 1 Department of Neurological and Neurosurgical Nursing, Faculty of Health Science, Collegium Medicum in Bydgoszcz, Nicolaus Copernicus University in Toruń, 13-15 Jagiellońska St., 85-067 Bydgoszcz, Polandkarolinafilipskakf@gmail.com (K.F.-B.); 2 Department of Pedagogy, Kazimierz Wielki University, 30 Chodkiewicza St., 85-067 Bydgoszcz, Poland; 3 Department of Neurosurgery, Functional and Stereotactic Neurosurgery, Faculty of Health Science, Collegium Medicum in Bydgoszcz, Nicolaus Copernicus University in Toruń, 13-15 Jagiellońska St., 85-067 Bydgoszcz, Poland; pawel.sokal@cm.umk.pl

**Keywords:** aneurysmal subarachnoid hemorrhage, inflammatory blood cell ratios, neutrophil-to-lymphocyte ratio, functional outcome, prognostic markers

## Abstract

**Background/Objectives**: Early functional status at hospital discharge is a clinically relevant outcome after aneurysmal subarachnoid hemorrhage (aSAH), but early prognostic assessment remains challenging. We evaluated whether admission inflammatory blood cell ratios were associated with discharge independence and added prognostic information beyond established neurological severity scales. **Methods**: In this retrospective single-center cohort study, 252 consecutive adults with aSAH were screened, and 144 endovascularly treated patients with available admission complete blood count with differential were included. Discharge independence was defined as a Barthel Index score ≥60 at hospital discharge. A clinical reference model included age, World Federation of Neurosurgical Societies (WFNS) grade, and Hunt–Hess grade. Multivariable logistic regression was used to assess associations between inflammatory ratios and discharge independence. Discrimination was assessed using receiver operating characteristic analysis with DeLong’s test, and the final model was internally validated by bootstrap resampling. **Results**: Forty-one patients (28.5%) achieved discharge independence. Higher admission neutrophil-to-lymphocyte ratio (NLR) was independently associated with lower odds of discharge independence (adjusted odds ratio 0.47 per interquartile range increase, 95% CI 0.24–0.90; *p* = 0.022). Adding NLR to the clinical reference model improved discrimination (AUC 0.790 vs. 0.737; *p* = 0.039), with an optimism-corrected AUC of 0.767 after bootstrap validation. Other inflammatory indices did not significantly improve discrimination. **Conclusions**: In this single-center retrospective cohort of endovascularly treated patients with aSAH, admission NLR was independently associated with discharge independence and provided modest incremental prognostic information beyond established neurological severity scales.

## 1. Introduction

Aneurysmal subarachnoid hemorrhage (aSAH) remains a severe neurological emergency associated with substantial morbidity and mortality despite major advances in neurocritical and neurosurgical care. Contemporary reviews emphasize that aSAH continues to carry a high burden of death and disability, with persistent deficits in function, cognition, behavior, and quality of life among survivors [1,2]. Mortality has nevertheless declined over recent decades, likely reflecting advances in aneurysm securement, endovascular techniques, and specialized neurocritical care [1,2,3].

Long-term neurological and functional impairment nevertheless remains common among survivors. Up to one-third of patients experience decreased quality of life, cognitive dysfunction, depression, and limitations in activities of daily living [2,4]. Five-year mortality may reach 29%, and poor functional outcome is reported in more than one-third of patients [5,6]. Functional recovery after aSAH is heterogeneous, and many survivors require prolonged hospitalization, institutional care, or intensive rehabilitation [5,6].

Functional status at hospital discharge is, therefore, a clinically relevant early outcome. It reflects not only the severity of the initial hemorrhage but also the cumulative effects of early brain injury, systemic complications, and in-hospital management, while directly influencing discharge planning and early rehabilitation strategies [2,7]. However, early identification of patients at risk for poor discharge independence remains challenging. Established clinical grading systems, such as the Hunt–Hess scale [8] and the World Federation of Neurosurgical Societies (WFNS) scale [9], primarily capture neurological severity at presentation and may not fully reflect systemic processes that influence short-term recovery during the acute phase [1,7].

Systemic inflammation is increasingly recognized as an important contributor to secondary brain injury after aSAH [2]. Experimental and clinical studies show that early inflammatory responses involve activation of innate immune pathways, disruption of the blood–brain barrier, cytokine release, and leukocyte recruitment, all of which have been linked to early brain injury and delayed cerebral ischemia [10,11,12]. These inflammatory cascades extend beyond the central nervous system. They are mirrored by peripheral immune responses, including dynamic alterations in circulating leukocyte populations and inflammatory mediators during the acute phase of aSAH [11,12,13].

In this context, inflammatory blood cell ratios derived from routine complete blood counts, including the neutrophil-to-lymphocyte ratio (NLR), lymphocyte-to-monocyte ratio (LMR), platelet-to-lymphocyte ratio (PLR), and systemic immune-inflammation index (SII), have emerged as accessible markers of systemic inflammatory activation [14,15,16]. These indices are inexpensive, widely available, and biologically plausible, as they reflect the balance between pro-inflammatory and regulatory immune components. Several observational studies and meta-analyses have reported associations between elevated inflammatory ratios, particularly NLR and SII, and adverse outcomes such as delayed cerebral ischemia, poor functional recovery, and mortality [15,16,17,18,19].

However, the available evidence remains methodologically heterogeneous. Many studies focus on mortality or selected complications rather than early functional independence, frequently use different biomarker cut-offs, and often do not comprehensively evaluate whether inflammatory ratios add prognostic information beyond established neurological severity scales [12,13,14,15,16,17,18,19]. In addition, internally validated multivariable analyses comparing several inflammatory blood cell ratios within the same cohort remain limited. As a result, the independent association of admission inflammatory ratios with discharge independence after aSAH, as well as their incremental prognostic value beyond routine clinical assessment, remains insufficiently defined.

Therefore, the aim of this study was to evaluate the association between admission inflammatory blood cell ratios and discharge independence at hospital discharge in patients with aneurysmal subarachnoid hemorrhage and to assess whether these indices provide incremental prognostic information beyond established neurological severity scales.

## 2. Materials and Methods

### 2.1. Study Design and Setting

This retrospective observational cohort study was conducted at the Department of Neurosurgery at the University Hospital, a tertiary referral center that provides neurosurgical and neurocritical care. Routinely collected clinical documentation and electronic medical records were reviewed for the period from 1 January 2020 to 31 December 2024.

### 2.2. Study Population and Eligibility Criteria

All consecutive adult patients hospitalized during the study period with a principal diagnosis consistent with aneurysmal subarachnoid hemorrhage (aSAH), operationalized as ICD-10 codes I60.0–I60.9 [20], were screened. During the study period, 252 patients were screened. Of these, 144 patients with an available admission complete blood count with differential (CBC-D) were included in the final analytic cohort. Patients without admission CBC-D were excluded because inflammatory indices could not be calculated. Missing admission CBC-D reflected routine clinical practice rather than predefined clinical exclusion criteria.

The inclusion criteria were as follows: diagnosis of aSAH (ICD-10 I60.0–I60.9), hospitalization at the study center between 2020 and 2024, availability of admission CBC-D for inflammatory index calculation, documented functional assessment at hospital discharge using the Barthel Index [21], and endovascular aneurysm treatment during the index hospitalization. All included patients were treated endovascularly. The exclusion criteria were non-aneurysmal or traumatic subarachnoid hemorrhage, missing admission CBC-D precluding inflammatory index calculation, missing discharge Barthel Index, conservative management, or primary surgical clipping without endovascular treatment, in order to maintain a therapeutically homogeneous cohort.

To assess potential selection bias related to missing admission CBC-D, baseline characteristics of included and excluded patients were compared using appropriate statistical tests.

### 2.3. Data Sources and Variables

Data were abstracted from electronic medical records, including admission notes, nursing documentation, imaging reports, and laboratory results. Data abstraction was performed by trained study personnel using a standardized data collection form to ensure consistent recording of variables.

Collected variables included demographics (age and sex), comorbidities, and admission severity measures, including the World Federation of Neurosurgical Societies (WFNS) grade [9], Hunt–Hess grade [8], and Glasgow Coma Scale (GCS) score [22]. Nutritional risk screening (NRS-2002) was recorded when available as part of routine care [23]. Treatment-related variables, including external ventricular drainage, decompressive craniectomy, aneurysm treatment modality, and length of hospital stay, were also extracted.

Length of hospital stay was defined as the number of days from hospital admission to discharge or in-hospital death during the index hospitalization.

### 2.4. Inflammatory Indices

Admission peripheral blood samples obtained as part of routine clinical care were used to calculate systemic inflammatory indices derived from CBC-D. These included the neutrophil-to-lymphocyte ratio (NLR), calculated as the absolute neutrophil count divided by the absolute lymphocyte count; the lymphocyte-to-monocyte ratio (LMR), calculated as the absolute lymphocyte count divided by the absolute monocyte count; the platelet-to-lymphocyte ratio (PLR), calculated as the platelet count divided by the absolute lymphocyte count; and the systemic immune-inflammation index (SII), calculated as platelet count × absolute neutrophil count/absolute lymphocyte count [24,25,26,27].

Inflammatory indices were primarily modeled as continuous variables in order to preserve prognostic information.

### 2.5. Outcome Definition

The primary outcome was discharge independence, defined as a Barthel Index score of at least 60 at hospital discharge. Patients who died during hospitalization were classified as not discharge independent, as death precludes functional independence at discharge.

Hospital-acquired infections and in-hospital mortality were summarized descriptively. Comparisons of inflammatory indices according to hospital-acquired infection status and survival status were performed for descriptive purposes only. They were not included as covariates in the primary multivariable models because these events may lie on the causal pathway between early systemic inflammation and functional outcome.

### 2.6. Statistical Analysis

Continuous variables are presented as medians with interquartile ranges (IQRs), and categorical variables as counts with percentages. Between-group comparisons (discharge independent versus not discharge independent) were performed using the Mann–Whitney U test for continuous variables and the chi-square test or Fisher’s exact test for categorical variables, as appropriate.

To evaluate associations with discharge independence, multivariable logistic regression models were constructed. A prespecified clinical reference model included age, WFNS grade, and Hunt–Hess grade as established measures of admission severity. Potential collinearity between WFNS and Hunt–Hess grades was evaluated before inclusion in multivariable models and was found to be high (Spearman’s ρ = 0.918, *p* < 0.001). Both scores were nevertheless retained in the prespecified clinical reference model to preserve comparability with the prior literature and routine clinical assessment. Given this strong collinearity, the individual regression coefficients for WFNS and Hunt–Hess should not be interpreted as independent effects and are presented primarily as components of the prespecified clinical reference model. As a sensitivity analysis, additional adjusted models, including only one neurological severity scale at a time (WFNS or Hunt–Hess), were fitted to assess whether the association between NLR and discharge independence remained consistent despite the high collinearity between these two severity measures. Variance inflation factors were also examined to further characterize collinearity within the primary model. WFNS and Hunt–Hess were modeled as ordinal variables, with 1-point increases, whereas age was modeled per 10-year increase.

Each inflammatory index (NLR, LMR, PLR, and SII) was examined in a separate adjusted model by adding the marker individually to the clinical reference model to avoid collinearity among the inflammatory parameters. Inflammatory indices were modeled as continuous variables scaled per IQR increase to facilitate interpretability and comparability across markers. Distribution of inflammatory indices was assessed before modeling, and the assumption of linearity in the logit for continuous variables was evaluated graphically. Interaction terms were not evaluated because the modest sample size and number of outcome events did not support reliable interaction modeling without substantial risk of overparameterization. Adjusted odds ratios (ORs) with 95% confidence intervals (CIs) were reported.

Analyses were performed using complete-case data; no imputation of missing values was conducted.

Model discrimination was quantified using the area under the receiver operating characteristic curve (AUC) based on predicted probabilities from each multivariable model. Incremental discriminative performance beyond the clinical reference model was assessed using DeLong’s test for correlated receiver operating characteristic curves. AUC 95% confidence intervals were estimated using nonparametric bootstrap resampling (2000 iterations).

Internal validation of the final multivariable model, including age, WFNS grade, Hunt–Hess grade, and NLR, was performed using bootstrap resampling (1000 iterations). With 41 discharge-independent patients and four predictors in the final model, the approximate events-per-variable ratio was 10.25; therefore, model performance was interpreted cautiously despite internal bootstrap validation.

Model optimism was estimated as the mean difference between model performance in bootstrap samples and performance of the bootstrap-fitted model when applied to the original dataset. Optimism-corrected estimates of discrimination (AUC), calibration slope, calibration intercept, and Brier score were obtained by subtracting optimism from apparent model performance. Calibration was additionally assessed graphically using a calibration plot based on deciles of predicted risk. All bootstrap procedures were performed with a fixed random seed to ensure reproducibility.

An exploratory receiver operating characteristic analysis was performed to derive an admission NLR cut-off for discharge non-independence using the Youden index. This threshold was considered hypothesis-generating and requires external validation.

To assess potential selection bias related to missing admission CBC-D, baseline characteristics of included and excluded patients were compared using the Mann–Whitney U test for continuous and ordinal variables and Fisher’s exact test for categorical variables.

All tests were two-sided, and *p*-values < 0.05 were considered statistically significant. Statistical analyses were performed using the R statistical environment (version 3.6.0 or later) in RStudio (version 2026.01.1+403; Posit Software, Boston, MA, USA). The study was conducted and reported in accordance with the TRIPOD statement for prediction model development and validation and the STROBE recommendations for observational cohort studies.

## 3. Results

### 3.1. Cohort Characteristics and Univariable Comparisons

**Figure 1:**
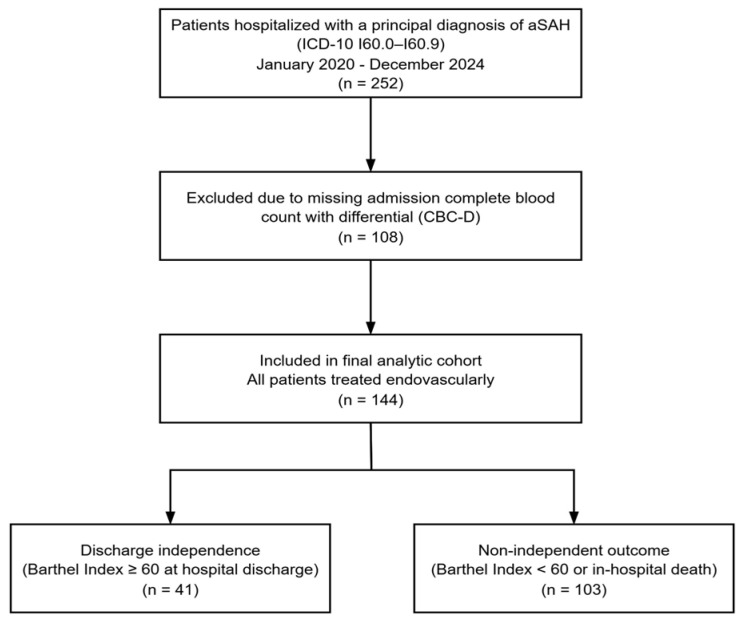
Flowchart of patient selection. Flowchart showing screening of patients with aneurysmal subarachnoid hemorrhage (aSAH), exclusion due to missing admission complete blood count with differential (CBC-D), and the final analytic cohort stratified by discharge independence at hospital discharge.

Patient selection is shown in Figure 1.

During the study period, 252 patients with aneurysmal subarachnoid hemorrhage were screened; 144 were included in the final analytic cohort, whereas 108 were excluded because admission complete blood count with differential (CBC-D) was unavailable. In excluded patients, only a standard blood count without differential was obtained at admission, which precluded calculation of inflammatory indices. Baseline characteristics of included and excluded patients are presented in Supplementary Table S3. No statistically significant differences were observed between groups in age, sex, WFNS grade, Hunt–Hess grade, Glasgow Coma Scale score, or in-hospital mortality (all *p* > 0.05).

Among the 144 included patients, 41 (28.5%) achieved discharge independence (Barthel Index ≥ 60) at hospital discharge, whereas 103 (71.5%) did not. Patients who achieved discharge independence were younger (median 55 vs. 62 years; *p* = 0.031) and less frequently female (43.9% vs. 68.0%; *p* = 0.013). Admission neurological severity also differed significantly between groups. Patients who achieved discharge independence presented with lower WFNS scores (median 1 [IQR 1–2] vs. 2 [1–5]; *p* < 0.001), lower Hunt–Hess grades (1 [1–2] vs. 3 [2–4]; *p* < 0.001), and higher Glasgow Coma Scale scores at admission (15 [15–15] vs. 13 [6–15]; *p* < 0.001) (Table 1).

Admission inflammatory indices also differed according to discharge independence status. Compared with patients who did not achieve discharge independence, those who achieved discharge independence had lower neutrophil-to-lymphocyte ratio (4.3 [2.5–6.5] vs. 9.2 [5.6–13.0]; *p* < 0.001), lower platelet-to-lymphocyte ratio (143.4 [105.4–211.7] vs. 186.5 [138.7–281.5]; *p* = 0.010), and lower systemic immune-inflammation index (1061.1 [686.9–1702.9] vs. 1892.3 [1204.1–3000.7]; *p* < 0.001), whereas lymphocyte-to-monocyte ratio was higher among patients who achieved discharge independence (2.09 [1.35–3.40] vs. 1.65 [1.01–2.49]; *p* = 0.024) (Table 1).

### 3.2. Hospital-Acquired Infections and In-Hospital Mortality

Hospital-acquired infections occurred in 12 of 144 patients (8.3%). In-hospital mortality was observed in 19/144 patients (13.2%), and all deaths occurred in the non-independent group (0% vs. 18.4%; *p* < 0.001). Admission inflammatory indices did not differ significantly according to hospital-acquired infection status or in-hospital mortality (all *p* > 0.05). However, the small number of events limited statistical precision, and point estimates suggested higher NLR and SII values among patients with hospital-acquired infection (Supplementary Table S1).

### 3.3. Multivariable Associations with Discharge Independence

In the prespecified clinical reference model including age, WFNS score, and Hunt–Hess grade, increasing age was inversely associated with discharge independence (OR per 10-year increase 0.77, 95% CI 0.58–1.02; *p* = 0.065). WFNS and Hunt–Hess were not independently associated with discharge independence, suggesting overlap in the clinical severity captured by these scales (Table 2).

When inflammatory indices were added individually to the clinical reference model and scaled per interquartile range, a higher admission neutrophil-to-lymphocyte ratio was associated with lower odds of achieving discharge independence (adjusted OR 0.47 per IQR increase [IQR 7.33], 95% CI 0.24–0.90; *p* = 0.022). In contrast, the lymphocyte-to-monocyte ratio was not independently associated with discharge independence (OR 1.34 per IQR [IQR 1.74], 95% CI 0.85–2.12; *p* = 0.203). Platelet-to-lymphocyte ratio showed a borderline inverse association that did not reach statistical significance (OR 0.56 per IQR [IQR 140.22], 95% CI 0.30–1.04; *p* = 0.065). Similarly, systemic immune-inflammation index was not independently associated with discharge independence (OR 0.62 per IQR [IQR 1815.8], 95% CI 0.36–1.07; *p* = 0.086), although the direction of effect was consistent with worse functional outcome at higher levels of systemic inflammation (Table 2).

In sensitivity analyses including only one neurological severity scale at a time, the association between NLR and discharge independence remained broadly consistent. In the model including age, WFNS, and NLR, higher NLR remained associated with lower odds of discharge independence (OR 0.47 per IQR increase, 95% CI 0.25–0.89; *p* = 0.021; AUC 0.791). Similarly, in the model including age, Hunt–Hess, and NLR, higher NLR remained associated with lower odds of discharge independence (OR 0.49 per IQR increase, 95% CI 0.26–0.91; *p* = 0.025; AUC 0.778). Variance inflation factors in the primary model were elevated for WFNS and Hunt–Hess (7.10 and 7.15, respectively), supporting substantial collinearity between these variables (Supplementary Table S5).

### 3.4. Incremental Prognostic Information Beyond Clinical Severity Scales

The clinical reference model incorporating age, WFNS score, and Hunt–Hess grade yielded an AUC of 0.737 (bootstrap 95% CI 0.650–0.815) for discharge independence. The addition of the neutrophil-to-lymphocyte ratio was associated with higher discrimination (AUC 0.790, 95% CI 0.700–0.867), corresponding to a ΔAUC of 0.053 with a significant DeLong test (*p* = 0.039). In contrast, inclusion of lymphocyte-to-monocyte ratio, platelet-to-lymphocyte ratio, or systemic immune-inflammation index did not significantly improve discrimination beyond the clinical reference model (all *p* > 0.05) (Table 3).

Internal bootstrap validation (1000 resamples) of the final multivariable model, including age, WFNS score, Hunt–Hess grade, and NLR, yielded an optimism-corrected AUC of 0.767. The calibration slope was 0.861, the calibration intercept was −0.088, and the optimism-corrected Brier score was 0.171 (Table 4). The optimism-corrected calibration slope below 1 suggests mild overfitting, whereas the near-zero calibration intercept indicates limited systematic over- or underprediction. The optimism-corrected Brier score reflects overall prediction error and should be interpreted together with discrimination and calibration, with lower values indicating better predictive accuracy.

**Figure 2:**
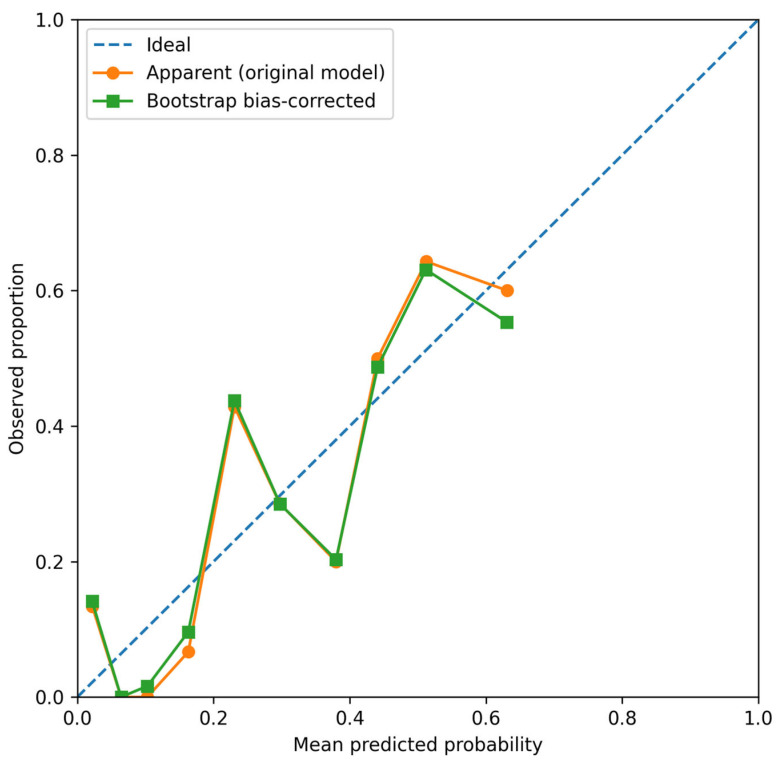
Calibration plot for the multivariable model predicting discharge independence. Calibration plot based on deciles of predicted probability for the model, including age, WFNS grade, Hunt–Hess grade, and neutrophil-to-lymphocyte ratio (NLR). The dashed line represents ideal calibration. The orange line shows the apparent model performance, and the green line shows bootstrap-bias-corrected calibration based on 1000 resamples. The x-axis represents the mean predicted probability, and the y-axis the observed proportion of discharge independence.

The corresponding calibration plot is shown in Figure 2.

The full specification of the final multivariable logistic regression model, including the intercept and regression coefficients for age, WFNS grade, Hunt–Hess grade, and admission neutrophil-to-lymphocyte ratio, is provided in Supplementary Table S4.

### 3.5. Exploratory NLR Cut-Off Analysis

In exploratory receiver operating characteristic analysis with discharge non-independence as the positive class, the optimal admission NLR cut-off was 5.39 (Youden index), yielding a sensitivity of 0.78 and a specificity of 0.66 (AUC 0.71). Bootstrap resampling indicated moderate variability of this threshold (approximately 4.39–9.22), supporting its interpretation as hypothesis-generating, cohort-specific, and not suitable for clinical application without requiring external validation (Supplementary Table S2). Accordingly, continuous modeling of NLR remained the primary analytic approach in the present study.

## 4. Discussion

In this retrospective single-center cohort of endovascularly treated patients with aneurysmal subarachnoid hemorrhage, admission NLR was independently associated with discharge independence and provided modest incremental prognostic information beyond a clinical reference model based on age, WFNS grade, and Hunt–Hess grade. In contrast, LMR, PLR, and SII did not demonstrate comparable added prognostic value after adjustment. Internal bootstrap validation indicated moderate model stability, although some optimism was present, and the findings should be interpreted in light of the modest sample size and limited number of outcome events.

These findings are consistent with previous reports showing that elevated admission NLR is associated with unfavorable short-term outcomes after aSAH and may capture prognostic information beyond neurological severity alone [14,15,16,28,29]. Our results extend the current literature by focusing on early discharge independence, comparing several inflammatory blood cell ratios within the same cohort, and evaluating their added prognostic contribution beyond routinely used clinical severity scales in an internally validated multivariable framework. This is relevant because many earlier studies emphasized mortality, delayed cerebral ischemia, or selected complications rather than early functional status at hospital discharge [14,16,29].

Discharge independence was defined as a Barthel Index score ≥60, a threshold commonly interpreted as indicating at least moderate functional capacity [21,30,31]. This outcome was selected because it reflects early recovery in a clinically meaningful way and has direct implications for discharge planning, rehabilitation needs, and post-acute care pathways [30]. In this study, the threshold was intended to capture a level of function compatible with at least partial independence in basic activities of daily living at hospital discharge. In this context, the association between higher admission NLR and lower odds of discharge independence suggests that systemic inflammatory activation may be linked not only to neurological complications but also to early functional recovery [32].

From a pathophysiological perspective, elevated NLR may reflect heightened innate immune activation, stress-related lymphopenia, and a systemic inflammatory response, all of which contribute to secondary brain injury after aSAH [14,31,33]. Similar associations between elevated NLR and adverse outcomes have been reported in other cerebrovascular conditions, including ischemic stroke [34]. In aSAH, such mechanisms are biologically plausible because early inflammatory cascades are involved in blood–brain barrier dysfunction, microvascular disturbance, and delayed secondary injury. The persistence of NLR as an independent predictor after adjustment suggests that it may capture prognostic information not fully reflected by clinical severity scales alone.

By contrast, LMR, PLR, and SII did not retain independent associations in adjusted models and did not significantly improve discrimination. The lack of independent associations for WFNS and Hunt–Hess should also be interpreted in light of the strong correlation between these severity scales, which may have reduced the stability and interpretability of their individual coefficient estimates. Accordingly, the reference model should be interpreted primarily as a pragmatic clinical adjustment model rather than as a framework for comparing the independent prognostic contributions of these two highly correlated severity scales. Sensitivity analyses including only one severity scale at a time yielded broadly consistent results for NLR, supporting the robustness of the primary association. In our cohort, LMR, PLR, and SII may have been more strongly influenced by age and baseline neurological severity or may have been less robust markers of the specific inflammatory processes relevant to early functional recovery. Although this does not exclude their potential relevance in other cohorts, their role appeared less consistent than that of NLR in the present analysis.

Hospital-acquired infections and in-hospital mortality occurred predominantly among patients without discharge independence. These variables were analyzed descriptively and not included in the primary multivariable models because they may represent intermediate events along the pathway between early systemic inflammation and functional outcome, and their inclusion could have introduced over-adjustment bias [35,36]. Although inflammatory indices were numerically higher among patients with complications, the low number of events precluded firm conclusions. Nutritional risk and length of hospital stay were also not included in prognostic models because they may represent downstream or intermediate factors rather than baseline predictors of discharge independence [37]. The longer hospital stay observed among discharge-independent patients should be interpreted cautiously, because deaths in the non-independent group may have shortened hospitalization time and limited the interpretability of length of stay as a prognostic variable.

From a clinical perspective, admission NLR may serve as a supportive adjunctive marker in the early prognostic assessment of patients with aSAH. However, the observed improvement in discrimination was modest (ΔAUC = 0.053), and this study did not assess whether adding NLR changes bedside decision-making or improves outcomes. Accordingly, the present results support incremental prognostic relevance rather than established clinical utility [38]. External validation remains necessary.

### Limitations

Several limitations should be acknowledged. First, the retrospective single-center design limits generalizability and precludes causal inference. Second, selection bias related to laboratory data availability should be considered: of 252 consecutive patients screened, 144 had admission complete blood count with differential available and were included in the analysis. The missing admission CBC-D reflected routine clinical practice rather than predefined exclusion criteria; however, non-random missingness cannot be excluded. Potential mechanisms may have included organizational, logistical, or timing-related factors affecting the availability of differential counts at admission. Although baseline characteristics of included and excluded patients were broadly comparable (Supplementary Table S3), residual selection bias and limited generalizability remain possible. Third, the sample size was modest, particularly with respect to the number of outcome events. Although the final model included 41 discharge-independent patients for four predictors, corresponding to an approximate events-per-variable ratio of 10.25, some risk of model instability and overfitting cannot be excluded despite internal bootstrap validation.

Fourth, inflammatory indices were measured only once at hospital admission. Serial measurements might better reflect inflammatory dynamics and provide stronger prognostic information. Although blood samples were obtained at hospital admission, the exact interval from symptom onset to admission was not standardized in this retrospective study, and early treatment-related or peri-procedural factors may also have influenced inflammatory marker values. Fifth, the cohort was restricted to patients treated endovascularly in order to preserve therapeutic homogeneity; therefore, the findings may not be directly generalizable to surgically treated or mixed-treatment populations, in which clipping remains clinically relevant and treatment-related factors may influence early functional recovery. Finally, although the exploratory NLR cut-off may facilitate interpretation, its bootstrap variability indicates that it should be regarded as hypothesis-generating, cohort-specific, and not suitable for clinical application as a fixed threshold. Continuous modeling of NLR remained the primary analytic approach. External validation was not performed, and formal measures of clinical utility were not assessed.

## 5. Conclusions

Admission neutrophil-to-lymphocyte ratio was independently associated with discharge independence after aneurysmal subarachnoid hemorrhage. It provided modest incremental prognostic information beyond established neurological severity scales in this single-center retrospective cohort. In contrast, LMR, PLR, and SII did not demonstrate comparable added prognostic value in adjusted analyses. These findings support the relevance of early systemic inflammation to short-term functional recovery and suggest that admission NLR may serve as a supportive adjunctive prognostic marker. However, the observed incremental prognostic gain was modest, and its broader clinical utility remains uncertain. Prospective multicenter studies with external validation, serial biomarker assessment, and formal evaluation of decision-making impact are needed before clinical application can be more firmly established.

## Figures and Tables

**Table 1:** Baseline characteristics of patients with aneurysmal subarachnoid hemorrhage stratified by discharge independence at hospital discharge.

Variable	All Patients (n = 144)	Discharge Independent (Barthel ≥ 60) (n = 41)	Not Discharge Independent (Barthel < 60) (n = 103)	*p*-Value
Age, years	60 (46–67)	55 (44–63)	62 (47–70)	**0.031**
Female sex, n (%)	88 (61.1)	18 (43.9)	70 (68.0)	**0.013**
BMI, kg/m^2^ (available, n = 69)	26.7 (23.5–29.7)	26.9 (24.5–28.5)	26.2 (23.2–31.9)	0.986
Hypertension, n (%)	64 (44.4)	20 (48.8)	44 (42.7)	0.635
Diabetes mellitus, n (%)	17 (11.8)	4 (9.8)	13 (12.6)	0.779
WFNS grade	2 (1–4)	1 (1–2)	2 (1–5)	**<0.001**
Hunt–Hess grade	2 (1–4)	1 (1–2)	3 (2–4)	**<0.001**
GCS on admission	14 (8–15)	15 (15–15)	13 (6–15)	**<0.001**
NRS-2002 risk (≥3), n (%)	22 (15.3)	3 (7.3)	19 (18.4)	0.125
NLR	7.1 (4.4–11.8)	4.3 (2.5–6.5)	9.2 (5.6–13.0)	**<0.001**
LMR	1.70 (1.13–2.87)	2.09 (1.35–3.40)	1.65 (1.01–2.49)	**0.024**
PLR	185.5 (128.0–268.2)	143.4 (105.4–211.7)	186.5 (138.7–281.5)	**0.010**
SII	1722.7 (971.6–2787.5)	1061.1 (686.9–1702.9)	1892.3 (1204.1–3000.7)	**<0.001**
Length of hospital stay, days	3 (1–10)	8 (2–10)	2 (1–6.5)	**0.009**
Barthel Index on admission	25 (0–45)	45 (30–70)	5 (0–35)	**<0.001**

Notes: Values are presented as median (interquartile range, IQR) or number (percentage), as appropriate. Continuous variables were compared using the Mann–Whitney U test, and categorical variables were compared using the chi-squared test or Fisher’s exact test, as appropriate. Statistically significant *p*-values (*p* < 0.05) are shown in bold. Discharge independence was defined as a Barthel Index score ≥60 at hospital discharge. Abbreviations: BMI, body mass index; GCS, Glasgow Coma Scale; LMR, lymphocyte-to-monocyte ratio; NLR, neutrophil-to-lymphocyte ratio; NRS, Nutritional Risk Screening; PLR, platelet-to-lymphocyte ratio; SII, systemic immune-inflammation index; WFNS, World Federation of Neurosurgical Societies.

**Table 2:** Multivariable Logistic Regression Models for Discharge Independence.

Variable	Adjusted OR (95% CI)	*p*-Value
**Model 1 (clinical reference model)**		
Age (per 10 years)	0.77 (0.58–1.02)	0.065
WFNS grade (per 1-point increase)	0.63 (0.31–1.27)	0.192
Hunt–Hess grade (per 1-point increase)	0.87 (0.38–2.01)	0.747
**Model 2 (+NLR)**		
Age (per 10 years)	0.76 (0.57–1.02)	0.064
WFNS grade (per 1-point increase)	0.60 (0.29–1.24)	0.168
Hunt–Hess grade (per 1-point increase)	1.02 (0.43–2.41)	0.967
NLR (per IQR increase; IQR = 7.33)	0.47 (0.24–0.90)	**0.022**
**Model 3 (+LMR)**		
Age (per 10 years)	0.78 (0.59–1.04)	0.088
WFNS grade (per 1-point increase)	0.56 (0.27–1.15)	0.115
Hunt–Hess grade (per 1-point increase)	1.00 (0.43–2.34)	0.993
LMR (per IQR increase; IQR = 1.74)	1.34 (0.85–2.12)	0.203
**Model 4 (+PLR)**		
Age (per 10 years)	0.78 (0.59–1.04)	0.091
WFNS grade (per 1-point increase)	0.61 (0.30–1.25)	0.177
Hunt–Hess grade (per 1-point increase)	0.91 (0.40–2.09)	0.824
PLR (per IQR increase; IQR = 140.22)	0.56 (0.30–1.04)	0.065
**Model 5 (+SII)**		
Age (per 10 years)	0.75 (0.56–1.00)	**0.047**
WFNS grade (per 1-point increase)	0.62 (0.30–1.25)	0.180
Hunt–Hess grade (per 1-point increase)	0.97 (0.42–2.26)	0.946
SII (per IQR increase; IQR = 1815.8)	0.62 (0.36–1.07)	0.086

Notes: Adjusted odds ratios (ORs) with 95% confidence intervals (CIs) are reported. Odds ratios are adjusted for all covariates included in each model. Age is scaled per 10-year increase. Inflammatory markers are modeled as continuous variables and scaled per interquartile range (IQR) increase. WFNS and Hunt–Hess grades are modeled as ordinal variables (per 1-point increase). All models were fitted in the full study cohort (n = 144). Number of events (discharge independence): n = 41. Statistically significant *p*-values (*p* < 0.05) are shown in bold. Abbreviations: CI, confidence interval; IQR, interquartile range; LMR, lymphocyte-to-monocyte ratio; NLR, neutrophil-to-lymphocyte ratio; OR, odds ratio; PLR, platelet-to-lymphocyte ratio; SII, systemic immune-inflammation index; WFNS, World Federation of Neurosurgical Societies. The bold formatting reflects that the same variables (e.g., Age, WFNS grade, Hunt–Hess grade, PLR, etc.) were calculated for each model (Model 1–4). The bold values are used to highlight and visually distinguish the variables within each model.

**Table 3:** Incremental Prognostic Information of Inflammatory Markers Beyond Clinical Severity Scales.

Model	n	AUC	AUC (95% CI)	ΔAUC	*p* (DeLong)
Clinical model (age + WFNS + Hunt–Hess)	144	0.737	0.650–0.815	—	—
Clinical + NLR	144	0.790	0.700–0.867	0.053	**0.039**
Clinical + LMR	144	0.749	0.658–0.826	0.012	0.347
Clinical + PLR	144	0.763	0.677–0.846	0.026	0.248
Clinical + SII	144	0.764	0.674–0.847	0.027	0.178

Notes: AUCs were derived from predicted probabilities of the multivariable logistic regression models. Incremental discrimination was quantified as the change in AUC (ΔAUC) by comparing each extended model with the clinical reference model (age, WFNS, Hunt–Hess) using DeLong’s test for correlated ROC curves. AUC 95% confidence intervals were estimated using nonparametric bootstrap resampling (2000 iterations). All analyses were performed in the full study cohort (n = 144). Statistically significant *p*-values (*p* < 0.05) are shown in bold. Abbreviations: AUC, area under the receiver operating characteristic curve; CI, confidence interval; LMR, lymphocyte-to-monocyte ratio; NLR, neutrophil-to-lymphocyte ratio; PLR, platelet-to-lymphocyte ratio; ROC, receiver operating characteristic; SII, systemic immune-inflammation index; WFNS, World Federation of Neurosurgical Societies; ΔAUC, change in AUC.

**Table 4:** Internal Validation of the Final Multivariable Model for Discharge Independence.

Metric	Apparent Performance	Optimism-Corrected Performance
Area under the ROC curve (AUC)	0.790	0.767
Calibration slope	1.00	0.861
Calibration intercept	0.00	−0.088
Brier score	0.160	0.171

Notes: Internal validation of the multivariable logistic regression model was performed using bootstrap resampling with replacement (1000 iterations). The final model included age (per 10-year increase), WFNS grade (per 1-point increase), Hunt–Hess grade (per 1-point increase), and NLR (per interquartile range increase; IQR = 7.33). Optimism-corrected estimates were obtained by subtracting the average bootstrap optimism from the apparent model performance. Lower Brier score values indicate better model performance. Abbreviations: AUC, area under the receiver operating characteristic curve; ROC, receiver operating characteristic.

## Data Availability

The data presented in this study are available on request from the corresponding author. The data are not publicly available due to institutional data protection regulations and patient privacy restrictions.

## Supplementary Materials

The following supporting information can be downloaded at https://www.mdpi.com/article/10.3390/biomedicines14061186/s1. Supplementary Table S1: Hospital-acquired infections, mortality, and inflammatory markers in patients with aneurysmal subarachnoid hemorrhage; Supplementary Table S2: Exploratory neutrophil-to-lymphocyte ratio cut-off for predicting discharge non-independence after aneurysmal subarachnoid hemorrhage; Supplementary Table S3: Comparison of baseline characteristics between patients included in and excluded from the multivariable analysis; Supplementary Table S4: Full specification of the final multivariable logistic regression model for discharge independence; Supplementary Table S5: Sensitivity analyses and collinearity diagnostics for the primary multivariable model.

## Abbreviations

The following abbreviations are used in this manuscript:

aSAH	Aneurysmal subarachnoid hemorrhage
AUC	Area under the receiver operating characteristic curve
BMI	Body mass index
CBC-D	Complete blood count with differential
CI	Confidence interval
GCS	Glasgow Coma Scale
HAI	Hospital-acquired infection
IQR	Interquartile range
LMR	Lymphocyte-to-monocyte ratio
NLR	Neutrophil-to-lymphocyte ratio
NRS	Nutritional Risk Screening
OR	Odds ratio
PLR	Platelet-to-lymphocyte ratio
ROC	Receiver operating characteristic
SII	Systemic immune-inflammation index
WFNS	World Federation of Neurosurgical Societies
