# Supplementary material for: Admission Inflammatory Blood Cell Ratios as Prognostic Markers of Functional Outcome After Aneurysmal Subarachnoid Hemorrhage: A Single-Center Retrospective Cohort Study
Source: Biomedicines. 2026 May 24;14(6):1186. doi: 10.3390/biomedicines14061186 (PMC13296031; doi:10.3390/biomedicines14061186)
Supplement: Supplementary file 1 [file biomedicines-14-01186-s001.zip › biomedicines-4262432-supplementary.pdf]

Supplementary Table S1. Hospital-acquired infections, mortality, and inflammatory markers in patients with aneurysmal subarachnoid hemorrhage (n = 144).

A. HAI and in-hospital mortality according to discharge independence.

Variable	Overall (n = 144)	Barthel ≥60 (n = 41)	Barthel <60 (n = 103)	*p*-value†
Any hospital-acquired infection, n (%)	12 (8.3%)	2 (4.9%)	10 (9.7%)	0.507
Pneumonia, n (%)	6 (4.2%)	1 (2.4%)	5 (4.9%)	0.675
Urinary tract infection, n (%)	2 (1.4%)	0 (0.0%)	2 (1.9%)	1.000
CNS infection (ventriculitis), n (%)	2 (1.4%)	0 (0.0%)	2 (1.9%)	1.000
Bloodstream infection, n (%)	5 (3.5%)	1 (2.4%)	4 (3.9%)	1.000
In-hospital mortality, n (%)	19 (13.2%)	0 (0.0%)	19 (18.4%)	NA‡

B. Inflammatory markers according to HAI status.

Marker	No HAI (n = 132), median (IQR)	HAI (n = 12), median (IQR)	*p*-value§
NLR	6.7 (4.4–11.7)	9.8 (6.8–12.4)	0.306
LMR	1.71 (1.12–2.89)	1.57 (1.07–1.80)	0.412
PLR	185.0 (128.3–257.5)	201.6 (145.7–297.0)	0.420
SII	1685.3 (965.4–2671.3)	2193.9 (1766.4–2925.1)	0.164

C. Inflammatory markers according to in-hospital mortality status.

Marker	Survivors (n = 125), median (IQR)	Non-survivors (n = 19), median (IQR)	*p*-value§
NLR	6.7 (4.4–11.4)	11.1 (4.5–15.5)	0.283
LMR	1.67 (1.13–2.70)	1.75 (1.00–5.40)	0.565
PLR	184.5 (128.7–266.7)	185.5 (118.8–283.2)	0.904
SII	1685.5 (986.4–2598.0)	2027.8 (707.0–3266.9)	0.441

Notes: Values are n (%) or median (IQR). † Fisher's exact test (two-sided); ‡ Not formally tested because deaths were classified as not discharge independent by definition; § Mann–Whitney U test (two-sided); descriptive comparison only. Infection categories are not mutually exclusive. Due to low event counts, p-values for individual infection types should be interpreted cautiously. Abbreviations: CNS, central nervous system; HAI, hospital-acquired infection; IQR, interquartile range; LMR, lymphocyte-to-monocyte ratio; NLR, neutrophil-to-lymphocyte ratio; PLR, platelet-to-lymphocyte ratio; SII, systemic immune-inflammation index

Supplementary Table S2. Exploratory neutrophil-to-lymphocyte ratio cut-off for predicting discharge non-independence after aneurysmal subarachnoid hemorrhage.

Parameter	Value
Study cohort	Patients with aneurysmal subarachnoid hemorrhage (n = 144)
Outcome of interest	Discharge non-independence (Barthel Index <60 at hospital discharge)
Timing of NLR assessment	At hospital admission
Method for cut-off derivation	Receiver operating characteristic curve analysis
Cut-off selection criterion	Youden index (maximization of sensitivity + specificity − 1)
Optimal NLR cut-off	**5.39**
Sensitivity	0.78
Specificity	0.66
Positive predictive value (PPV)	0.85
Negative predictive value (NPV)	0.54
Area under the ROC curve (AUC)	0.71
Bootstrap resampling	2,000 iterations
95% bootstrap interval for cut-off	4.39–9.22

Notes: The NLR cut-off was derived in an exploratory analysis to enhance clinical interpretability. Because of moderate variability across bootstrap samples, this threshold should be considered cohort-specific and requires external validation. In the primary analyses, NLR was modeled as a continuous variable to preserve prognostic information and avoid loss of statistical power. Abbreviations: AUC, area under the receiver operating characteristic curve; NLR, neutrophil-to-lymphocyte ratio; NPV, negative predictive value; PPV, positive predictive value; ROC, receiver operating characteristic curve.

Supplementary Table S3. Comparison of baseline characteristics between patients included in and excluded from the multivariable analysis.

**Variable**	**Included (n = 144)**	**Excluded (n = 108)**	***p*-value**
Age, years	60 (46–67)	57 (45–62)	0.062
Female sex, n (%)	88 (61.1%)	68 (63.0%)	0.794
WFNS grade	2 (1–4)	2 (1–3)	0.051
Hunt–Hess grade	2 (1–3)	2 (1–2)	0.084
GCS	14 (10–15)	15 (13–15)	0.093
In-hospital mortality, n (%)	19 (13.2%)	9 (8.3%)	0.311

Notes: Values are median (IQR) or n (%). Baseline characteristics were compared between patients included in the multivariable analysis and those excluded because admission complete blood count with differential data were unavailable. Continuous and ordinal variables were compared using the Mann–Whitney U test, and categorical variables were compared using Fisher's exact test. All tests were two-sided, and a p-value < 0.05 was considered statistically significant. Abbreviations: GCS, Glasgow Coma Scale; IQR, interquartile range; WFNS, World Federation of Neurosurgical Societies.

Supplementary Table S4. Full specification of the final multivariable logistic regression model for discharge independence.

Predictor	Coding/scaling	β coefficient	SE	OR	95% CI	*p*-value
Intercept	—	2.515	0.961	12.36	1.88–81.34	0.009
Age	per 10-year increase	-0.271	0.146	0.76	0.57–1.02	0.064
WFNS grade	per 1-point increase	-0.511	0.370	0.60	0.29–1.24	0.168
Hunt–Hess grade	per 1-point increase	0.018	0.439	1.02	0.43–2.41	0.967
NLR	per IQR increase (IQR 7.33)	-0.758	0.331	0.47	0.24–0.90	0.022

Notes: β coefficients, standard errors (SEs), odds ratios (ORs), 95% confidence intervals (CIs), and p-values are shown for the final multivariable logistic regression model predicting discharge independence at hospital discharge. Age was modeled per 10-year increase, WFNS and Hunt–Hess grades per 1-point increase, and NLR per interquartile range (IQR) increase (IQR 7.33). ORs were calculated as exp(β). Predicted probability of discharge independence can be calculated as: $\text{logit}(p) = 2.515 - 0.271 \times \text{Age\_10} - 0.511 \times \text{WFNS} + 0.018 \times \text{Hunt–Hess} - 0.758 \times \text{NLR\_IQR}$, where Age_10 = age/10 and NLR_IQR = NLR/7.33; $p = \exp(\text{logit}(p)) / [1 + \exp(\text{logit}(p))]$. Abbreviations: CI, confidence interval; IQR, interquartile range; NLR, neutrophil-to-lymphocyte ratio; OR, odds ratio; SE, standard error; WFNS, World Federation of Neurosurgical Societies.

Supplementary Table S5. Sensitivity Analyses and Collinearity Diagnostics for the Primary Multivariable Model.

A. Collinearity diagnostics for the primary multivariable model.

Variable	VIF
Age (per 10-year increase)	1.01
WFNS grade	7.10
Hunt–Hess grade	7.15
NLR (per IQR increase; IQR = 7.33)	1.13

B. Sensitivity analyses using alternative adjusted models including only one neurological severity scale at a time.

Model	Variable	Adjusted OR (95% CI)	*p*-value	AUC
Age + WFNS + NLR	Age (per 10-year increase)	0.76 (0.57–1.02)	0.063	0.791
	WFNS grade (per 1-point increase)	0.61 (0.45–0.83)	0.002	
	NLR (per IQR increase; IQR = 7.33)	0.47 (0.25–0.89)	0.021	
Age + Hunt–Hess + NLR	Age (per 10-year increase)	0.76 (0.57–1.01)	0.055	0.778
	Hunt–Hess grade (per 1-point increase)	0.58 (0.41–0.84)	0.003	
	NLR (per IQR increase; IQR = 7.33)	0.49 (0.26–0.91)	0.025	

Notes: Variance inflation factors (VIFs) were calculated for the primary multivariable model including age, WFNS grade, Hunt–Hess grade, and NLR. Sensitivity analyses were performed using alternative adjusted logistic regression models including only one neurological severity scale at a time to assess whether the association between NLR and discharge independence remained consistent despite the high collinearity between WFNS and Hunt–Hess. Odds ratios are adjusted for all covariates included in the corresponding model. AUC values were derived from predicted probabilities of each model and refer to the overall discriminative performance of each multivariable model rather than to individual predictors. Abbreviations: AUC, area under the receiver operating characteristic curve; CI, confidence interval; IQR, interquartile range; NLR, neutrophil-to-lymphocyte ratio; OR, odds ratio; VIF, variance inflation factor; WFNS, World Federation of Neurosurgical Societies.
